# Alterations in blood glucose concentration in wild rodents, *Holochilus sciureus,* naturally infected with *Schistosoma mansoni*

**DOI:** 10.1590/S1984-29612022019

**Published:** 2022-03-28

**Authors:** João Gustavo Mendes Rodrigues, Maria Gabriela Sampaio Lira, Ranielly Araújo Nogueira, Gleycka Cristine Carvalho Gomes, Irlla Correia Lima Licá, Jeferson Kelvin Alves de Oliveira Silva, Guilherme Silva Miranda, Nêuton Silva-Souza

**Affiliations:** 1 Programa de Pós-graduação em Parasitologia, Universidade Federal de Minas Gerais – UFMG, Belo Horizonte, MG, Brasil; 2 Departamento de Química e Biologia, Universidade Estadual do Maranhão – UEMA, São Luís, MA, Brasil; 3 Programa de Pós-graduação em Ciências da Saúde, Universidade Federal do Maranhão – UFMA, São Luís, MA, Brasil; 4 Departamento de Biologia, Instituto Federal de Educação, Ciência e Tecnologia do Maranhão, São Raimundo das Mangabeiras, MA, Brasil

**Keywords:** Schistosomiasis, wild reservoir, glycemic homeostasis, liver pathology, Esquistossomose, reservatório silvestre, homeostase glicêmica, patologia hepática

## Abstract

The present study aimed to evaluate the changes in peripheral blood glucose concentrations induced by *Schistosoma mansoni* infection in *Holochilus sciureus* rodents, a wild reservoir of the parasite. Glucose concentration was measured in the plasma of blood samples using a colorimetric enzymatic test. Biological parameters and *S. mansoni* burden in each rodent were also verified and correlated with glucose concentrations. A total of 76 *H. sciureus* were captured, out of which 20 (26%) were infected with *S. mansoni* (n=13 males and n=7 females). Although the parasite burden was comparable between the sexes, blood glucose concentration was lower in infected males and almost unchanged in females. Furthermore, histopathological data revealed that male rodents had a greater hepatic granulomatous inflammatory reaction than females. In addition, we also confirmed that the weight and total length of the analyzed animals had no effect on glucose levels. Therefore, natural infection with *S. mansoni* in *H. sciureus* may have a lower impact on glycemic homeostasis in females, which will help us understand the role of these rodents as reservoirs of *S. mansoni*.

## Resumo

Neste presente estudo, objetivou-se avaliar as alterações induzidas pela infecção natural de *Schistosoma mansoni* nas concentrações de glicose do sangue periférico de roedores *Holochilus sciureus*, reservatório silvestre do parasito. A dosagem da concentração de glicose foi realizada no plasma de amostras de sangue por meio de teste enzimático colorimétrico. Parâmetros biológicos e carga parasitária de *S. mansoni* também foram verificados em cada roedor e correlacionados com as concentrações de glicose. Um total de 76 *H. sciureus* foram capturados, dentre os quais 20 (26%) estavam infectados por *S. mansoni* (n=13 machos e n=7 fêmeas). Apesar da carga parasitária ter sido semelhante entre os sexos, as concentrações de glicose no sangue foram menores em machos naturalmente infectados e quase inalteradas em fêmeas. Além disso, os dados histopatológicos mostraram uma maior reação inflamatória granulomatosa hepática em roedores machos, quando comparados com as fêmeas. Adicionalmente, também foi confirmado que o peso e o comprimento total dos animas analisados não interferiram nos valores de glicose. Portanto, a infecção natural por *S. mansoni* em *H. sciureus* pode induzir menos impacto na homeostase glicêmica em fêmeas, o que pode ajudar a melhor entender o papel desses roedores como reservatórios de *S. mansoni*.

## Introduction

Schistosomiasis is a neglected tropical disease caused by parasites of the genus *Schistosoma* (Trematoda, Schistosomatidae), affecting approximately 250 million people worldwide (Gryseels et al., 2006; Weerakoon et al., 2015; WHO, 2017). *Schistosoma mansoni* is widely distributed in Africa and South America, primarily in Brazil, and it is considered the main etiological agent of intestinal schistosomiasis (Gryseels et al., 2006; Weerakoon et al., 2015, McManus et al., 2018). The pathology of this disease is associated with the severe damage caused predominantly in the liver and intestine because of an intense inflammatory reaction induced by the soluble antigens released by parasite eggs trapped in the tissues (Lenzi et al., 1998; Pearce & MacDonald, 2002; Andrade, 2004; Hams et al., 2013).

Although *S. mansoni* is considered a typical human parasite, it has been found in wild rodents in several endemic areas worldwide (Théron et al., 1992; Miranda et al., 2017; Catalano et al., 2020). In Brazil, the semiaquatic rodents *Holochilus sciureus* and *Nectomys squamipes* are considered to be the main species potentially involved in *S. mansoni* transmission (Rey, 1993; Miranda et al., 2017) because they eliminate viable eggs in their feces and the infection does not affect the survival, mobility, and reproduction of these animals (Picot, 1992; D'Andrea et al., 2000). Thus, understanding the dynamics between the rodents’ high susceptibility to the parasite and the reduced negative effect of infection in these rodents is critical to understand their role in schistosomiasis transmission.

To better assess this issue, previous studies examined the liver function of *N. squamipes* during natural infection with *S. mansoni* and demonstrated constant glucose levels (Costa et al., 2013). Furthermore, the metabolism of these animals during natural infection allows for an excessive accumulation of lipids in the liver (steatosis), which may prevent intense severe liver inflammatory reactions (Amaral et al., 2016). In the case of *H. sciureus,* studies have shown that experimental infection with *S. mansoni* causes a decrease in glucose concentrations in younger infected animals (30 days old), but not in older infected animals (40 days old) (Bastos et al., 1985). However, no study has proposed a similar investigation during natural infections and comparatively between males and females of *H. sciureus* rodents as yet.

Thus, the present study aimed to evaluate the impact of *S. mansoni* on the blood glucose concentrations of naturally infected *H. sciureus* males and females.

## Material and Methods

### Study area and capture of *H. sciureus*

The captures were conducted in the city of São Bento, in the Baixada Ocidental of the Maranhão state, Brazil (02º41'45 “S 44º49'17” W). According to the Brazilian Ministry of Health (Brasil, 2018), the infection rate for *S. mansoni* in the human population is approximately 5% in this region.

From December 2014 to June 2015, *H. sciureus* rodents were captured using Tomahawk® traps (Jeetekno, Hazelhurst, WI, USA). Field collection was conducted according to the method given by do Carmo-Silva et al. (2019). Ten traps were distributed per collection point during the night, totaling 16 h (4 p.m. to 8 a.m.) in the field. A mixture of banana and peanut butter was used as bait. The traps were checked in the morning, and any other captured animals were immediately released. Each collection session lasted approximately two days. Rodent capture was authorized by the Biodiversity Authorization and Information System (n°40025/1).

### Biological aspects and blood collection

The captured *H. sciureus* rodents were immediately euthanized (Council of Ethics nº 05/2014/CEE/UEMA) with an anesthetic overdose (300 mg/kg of 5% ketamine and 30 mg/kg 2% xylazine hydrochloride) via the intraperitoneal route. Sex, weight, and total length (TL) were recorded (Lira et al., 2016). Blood was collected using a Pasteur pipette containing ethylenediaminetetraacetic acid (EDTA), from the axillary plexus vessels and stored in 5 mL vacutainer tubes (K3/KASVI®, Prime Cirúrgica, Cravinhos, SP, BRA). Tubes containing blood were centrifuged at 290 x g for 10 min at 4 °C. Plasma was collected and stored at -20 °C.

### *Schistosoma mansoni* burden

As described by Pellegrino & Siqueira (1956), a perfusion technique was used to confirm and quantify the parasite burden by *S. mansoni* in *H. sciureus*. In brief, a needle coupled to a perfusion pump (Automatic Pippeting Brewer Machine, model 60453, BD, Wazobia Enterprise, Houston, TX, USA) was inserted into the animals’ thoracic aorta for perfusion of the circulatory system with a 0.85% NaCl solution containing 80 U/L of heparin. The solution containing the adult worms was extravasated through the portal vein (previously sectioned) and collected individually from each animal in plastic beakers (600 mL). To clean the worms, the solution was washed with 0.85% NaCl solution several times. The worms were then differentiated into males and females (Neves et al., 1998) and counted using a stereomicroscope (Zeiss Stemi Dv4, Jena, Germany).

### Blood glucose concentration

To quantify the rodents’ blood plasma glucose levels, we used a commercially available kit from Bioclin® (Monoreagent Glucose, Quibasa Química Básica Ltda, Belo Horizonte, MG, BRA), following the manufacturer's instructions. Absorbances were read by using spectrophotometer (Bel Photonics, UV-M51) at a wavelength of 505 nm.

### Liver pathology

Histopathological analyses were carried out to examine the impact of natural *S. mansoni* infection on liver damage that could affect glucose metabolism. Liver samples (larger lobe) from each animal were stored in 10% formalin for 48 h and processed according to Luna (1968). After fixation, the samples were washed in tap water for 1h and placed in 70% ethanol. Then, all of the material was dehydrated for 1h in each increasing ethanol series (80, 90, 95, and 100%), clarified in xylol, embedded in paraffin for subsequent histological slide preparation (5-µm sections), and stained with hematoxylin and eosin. Qualitative histopathological analysis was performed using optical light microscopy (Liberio et al., 2011).

### Statistical analysis

Data were analyzed using Prism 8 software (GraphPad, San Diego, CA, USA). Results are presented as mean ± standard deviation. Normality was assessed using the Kolmogorov–Smirnov test. Data were analyzed using the student’s t-test or analysis of variance with the Bonferroni post-test. The relationship between blood glucose levels (variable response) and biometric parameters of rodents (co-variables) was determined using Pearson's correlation. Results were considered significant at p<0.05.

## Results

A total of 76 *H. sciureus* specimens were captured: 52 males (68%) and 24 females (32%). After parasitological evaluation, it was possible to identify 20 animals (26%) infected with *S. mansoni*. Among the naturally infected animals, thirteen were male (65%) and seven were female (35%). The parasite burden was similar between male and female rodents, with the mean number of worms ranging 6.7–7.6 (Table 1). In terms of *H. sciureus* biometrics, it was observed that male rodents are larger and heavier than females. Natural infection by *S. mansoni* did not significantly affect these parameters in either rodent sex (Table 2).

**Table 1:** Number of adult worms recovered from the circulatory system of *Holochilus sciureus* (male and female) naturally infected with *Schistosoma mansoni*, captured in the city of São Bento, Maranhão state, Brazil.

Parasites	Rodents		
	**Male (n=13)**	**Female (n=7)**	**p**^a^
Male worms	5.5 ±1.3	5.1 ±1.2	0.60
Female worms	2.1 ±0.8	1.6 ±0.8	0.21
Total	7.6 ±2.1	6.7 ±2.0	0.37

Data are presented as mean and standard deviation;

a p-value obtained by the Student’s t-test.

**Table 2:** Biometric parameters (weight and total length) of *Holochilus sciureus* (male and female) worm-negative (non-infected) and naturally infected with *Schistosoma mansoni*, captured in the city of São Bento, Maranhão state, Brazil.

Biological parameters	Male rodents			Female rodents		
	**Non-Infected (n=39)**	**Infected (n=13)**	**p^a^**	**Non-Infected (n=17)**	**Infected (n=7)**	**p**^a^
Weight (g)	207.5 ±58.6	223 ±66.9	0.47	136.9 ±31.8	145.8 ±46.1	0.59
Total length (cm)	33.2 ±2.6	34.3 ±3.6	0.30	29.7 ±2.2	30.7 ±2.9	0.34

Data are presented as mean and standard deviation;

a p-value obtained by the Student’s t-test.

**Figure 1:**
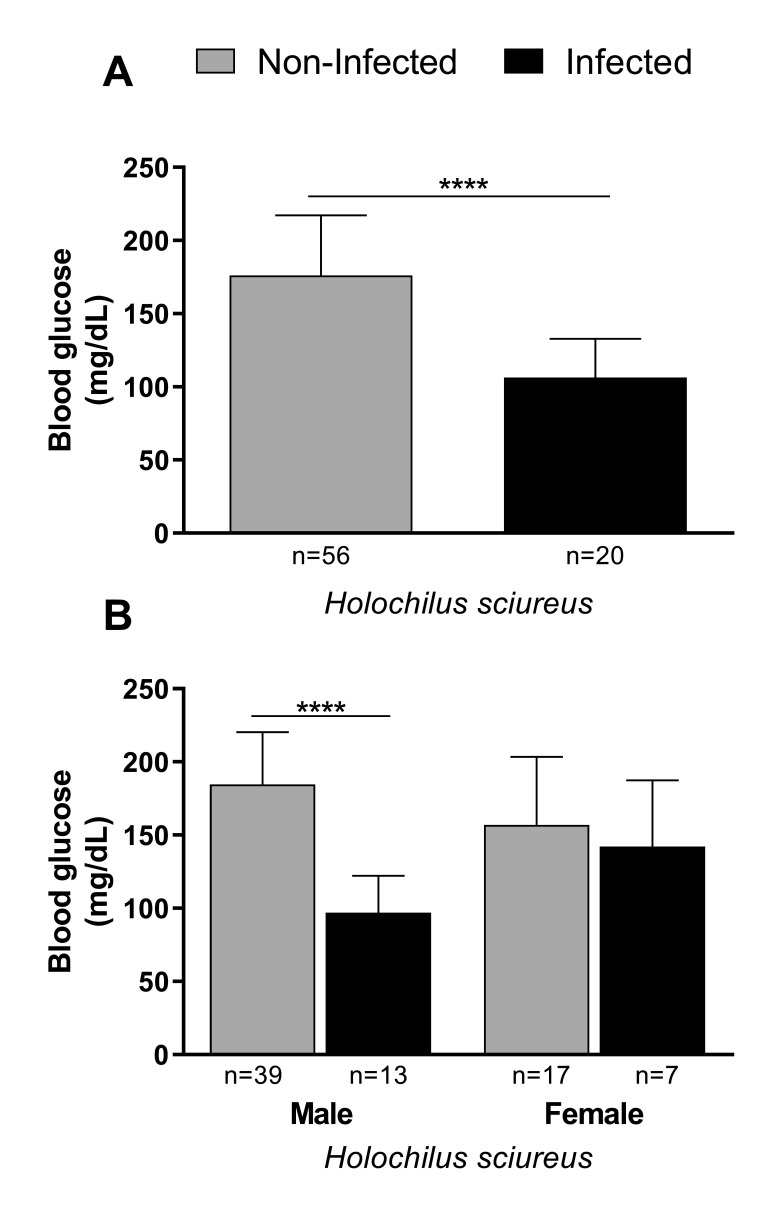
Glucose blood concentration in *Holochilus sciureus* non-infected (worm-negative), and naturally infected with *Schistosoma mansoni* (A). Comparison of glucose blood concentration in males and females of *H. sciureus*, infected or not (B). Data are expressed as mean and standard deviation and were analyzed by Student's t-test (in A) and by the one-way analysis of variance test (in B). ****Statistically significant.

**Figure 2:**
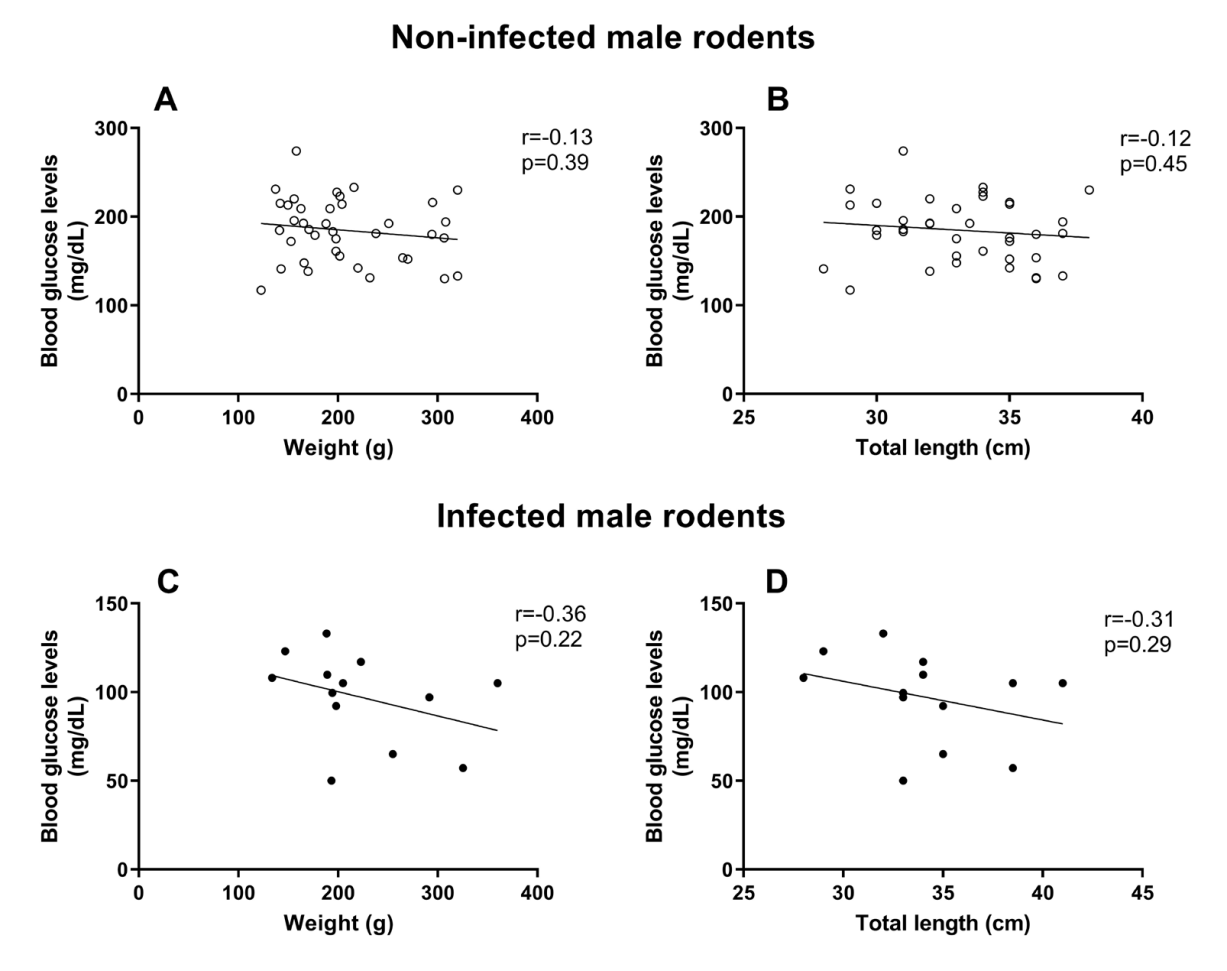
Correlation between blood glucose concentration and biometric parameters in male *Holochilus sciureus* non-infected (worm-negative) (A and B) and naturally infected with *Schistosoma mansoni* (C and D). Data were analyzed using Pearson correlation.

**Figure 3:**
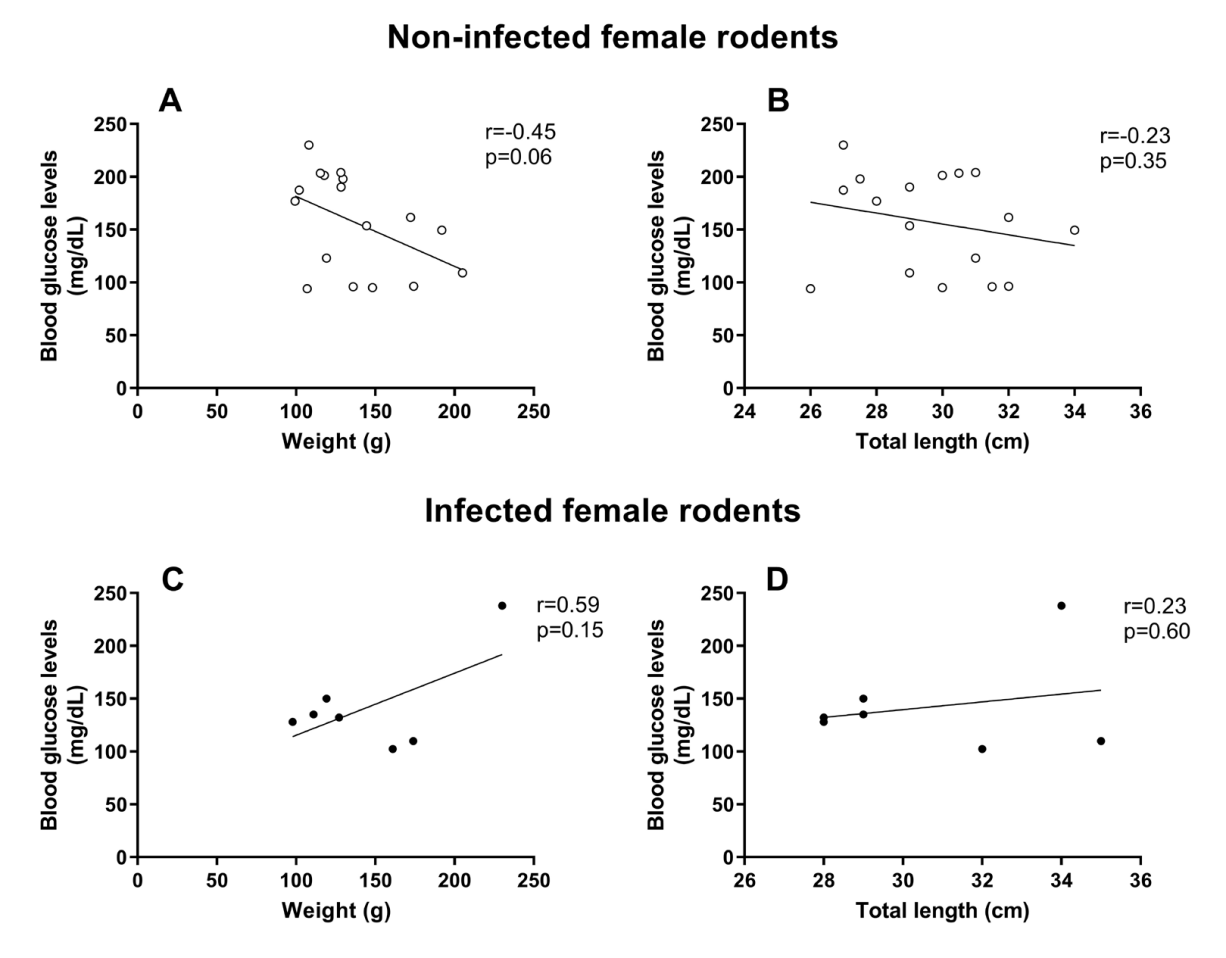
Correlation between blood glucose concentration and biometric parameters in female *Holochilus sciureus* non-infected (worm-negative) (A and B) and naturally infected with *Schistosoma mansoni* (C and D). Data were analyzed using Pearson correlation.

However, rodents, particularly males, naturally infected with *S. mansoni* showed a significant reduction in glucose concentration when compared to worm-negative animals (Figure 1). By examining the relationship between TL, weight, sex, and *S. mansoni* infection (covariates) versus glucose concentration (response variable), it was possible to observe that for males, all correlations were negative, but not significant (Figure 2). TL and weight were also negatively associated with glucose concentration in worm-negative females; however, in infected females, these biological parameters were positively associated with glucose in peripheral blood (with no significant difference) (Figure 3).

**Figure 4:**
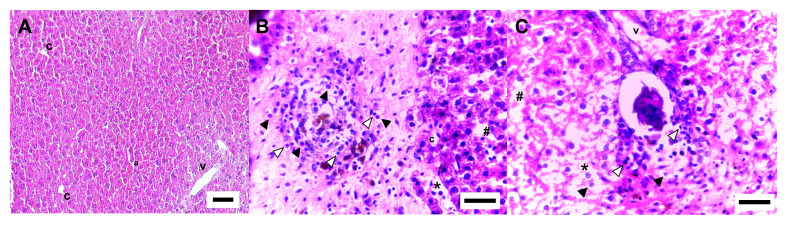
Representative photomicrograph of the liver parenchyma of *Holochilus sciureus* worm-negative (A), male (B), and female (C) naturally infected with *Schistosoma mansoni*. Histological slides were prepared using the large lobe liver of rodents, and 5 µm sections were stained with hematoxylin and eosin. a: arteriole, c: capillaries, v: venules, #: dilated sinusoids, *: loss of hepatocytes, black arrow: eosinophils, white arrow: lymphocytes. Scale bar = 20µm.

Histopathological analysis showed that the worm-negative animals had well-preserved liver tissue, with normal cell nuclei and cells without evidence of dysplastic and metaplastic processes or steatosis. The tissue showed free sinusoids, bile ducts, and vascular endothelium without alterations (Figure 4A). The histopathological evaluation of naturally infected males showed a liver tissue with intense inflammatory cell infiltration in the periovular region, forming the granuloma. Areas with parenchymal alterations, hepatocyte disappearance, and dilated sinusoids were also observed. The granuloma presented characteristics of acute-phase schistosomiasis and was rich in eosinophils. In addition, the granuloma had lymphocytes and collagen fibers that were poorly organized and had a loose appearance (Figure 4B). For naturally infected females, histopathological examination revealed the presence of areas with less hepatocytes and an apparent increase in the diameter of the sinusoids. In contrast, the granuloma had few inflammatory cells in the periovular region, in general lymphocytes, and few collagen fibers, characteristic of a more mature/modulated granuloma (Figure 4C).

## Discussion

In the present study, we demonstrated that natural infection by *S. mansoni* in *H. sciureus* might reduce the concentration of glucose in the peripheral blood of these rodents, particularly in males. Interestingly, even with a parasite burden comparable to that of males, infected females managed to maintain glucose concentration without major variations and better modulate the hepatic granulomatous reaction.

Schistosomiasis is primarily a human disease that causes severe tissue damage, mainly in the liver (Andrade, 2009; Hams et al., 2013; Lambertucci, 2014), affecting vital organ functions, such as the glucose production pathway (gluconeogenesis). Despite the tissue damage, studies on glucose concentrations in experimental models of induced obesity (Hussaarts et al., 2015; Duan et al., 2018; da Silva Filomeno et al., 2020) and in the human population (Duan et al., 2018; Wolde et al., 2019) have demonstrated that infection by *S. mansoni* or *S. japonicum* can improve glucose tolerance and reduce its concentration in the peripheral blood. Additionally, unisexual infections with *S. japonicum* (Duan et al., 2018) or only the inoculation of soluble egg antigens (SEA) of *S. mansoni* (Hussaarts et al., 2015) have shown to improve glycemic homeostasis in obese mice. The main mechanisms proposed to improve glucose tolerance and sensitivity are associated with the metabolism of adult schistosomes and the immune response induced by the infection. Adult worms can consume host glucose for survival (Bueding, 1950; Cornford et al., 1983), via the Embden–Meyerhof pathway (Bueding & MacKinnon, 1955; Fripp, 1972), which may contribute to lowering blood glucose levels. Furthermore, schistosomiasis infection induces a potent type 2 and regulatory immune response (Hesse et al., 2004; Burke et al., 2009; McManus et al., 2018) which can reduce chronic inflammation associated with metabolic disorders such as obesity (Hussaarts et al., 2015), improving homeostasis associated with glucose concentration. Thus, evaluating the relationship between schistosome development and host metabolism is essential for understanding the co-evolution of these two organisms (Saule et al., 2005). Based on this evidence, we conducted this study with naturally infected wild rodents *H. sciureus* to understand the compatibility of these animals with the *S. mansoni* parasite.

The rodent *H. sciureus* is considered the main wild reservoir of *S. mansoni* in Northeast Brazil, with natural infection rates ranging 18–30% (Rey, 1993; Miranda et al., 2017), which are higher than those of the local human population. In our study, we confirmed these findings, demonstrating that the natural infection rate in *H. sciureus* was 26%, reinforcing the need to understand the role of these animals in schistosomiasis transmission. In experimental infections with *H. sciureus*, high susceptibility and good tolerance to the infection have been demonstrated (Picot, 1992; Silva-Souza & Vasconcelos, 2005; Miranda et al., 2019). Moreover, it has been previously demonstrated that the blood glucose concentration of *H. sciureus* decreases progressively with the evolution of *S. mansoni* experimental infection, particularly in younger animals infected at 30 days old. However, animals infected at 40 days old did not show any major variation in blood glucose level and had a longer life span (Bastos et al., 1985), suggesting that the age of the animals, likely associated with the development of the immune system, contribute to a more balanced host-parasite relationship.

Glycemia assessments in wild rodents naturally infected with *S. mansoni,* on the other hand, have received little attention. Costa et al. (2013) conducted one of the few studies with this proposal, demonstrating that *N. squamipes* rodents with *S. mansoni* infection had similar serum and tissue glucose levels to non-infected ones, indicating a good tolerance to parasitism. However, data on parasite burden and rodent sex were not explored in these previous studies, and we believe that these parameters are critical for understanding the impact of *S. mansoni* infection on glycemia in these rodents. Thus, our data showed that females appear to be more tolerant to the infection, with similar plasma glucose values in infected and worm-negative animals (not infected). The preferential food of *H. sciureus* is composed of grass, seeds, and little invertebrates (Twice, 1962; Martino & Aguilera, 1993), and this diet may have some impact on glycemia in these animals. However, food preference and foraging behavior among *H. sciureus* males and females remain unclear and need to be investigated in further studies. Additionally, we also found that naturally infected females had a lower hepatic inflammatory response than male rodents, which may explain the glycemia almost unchanged in *H. sciureus* females. A previous experimental study (Silva-Souza & Vasconcelos, 2005) confirmed our data on natural infection by showing that females had a lower liver pathology than males. These data suggest that *H. sciureus* females naturally or experimentally infected with *S. mansoni* may induce a differential modulatory mechanism on the liver compared to males.

In summary, we have demonstrated for the first time that natural *S. mansoni* infections in *H. sciureus* rodents appear to have a lower impact on glycemia in females, as well as less liver damage when compared to naturally infected male rodents. Therefore, these findings may help us understand the true role of these animals in schistosomiasis.
